# Report of Medical Clinic

**Published:** 1856-02

**Authors:** J. G. Westmoreland

**Affiliations:** Professor of Materia Medica, &c., in the Atlanta Medical College


### ARTICLE III.

*Report of Medical Clinic, Continued.* By J. G. WESTMORELAND, M. D., Professor of Materia Medica, &c., in the Atlanta Medical College.

An interesting case of Hepatic Abscess in a negro man, aged about thirty, was subject to the examination of the Students for several weeks during the course of lectures, and was lectured upon and prescribed for in presence of the Class.

The subject of this case had been suffering most of the time during the Spring, with what was supposed to be some pectoral disease. This was inferred from the distressing cough which constantly harassed the patient; and not until he had entered the Infirmary, sometime in June, was the true nature of the disease known to his owner. The deceptiveness of the most prominent symptom in this case was well calculated to mislead the casual observer, and cause him not to suspect disease in the abdominal cavity. Hence the importance of critical examinations in all cases of disease, notwithstanding some of the leading symptoms may refer the difficulty to some particular organ.

In the case under consideration, the absence of disease of the lungs, ascertained by those almost unerring means, auscultation and percussion, and from positive symptoms of diseased liver, it was rational to conclude that all the difficulty was referable to the Hepatic derangement. The circumscribed enlargement and fluctuation which soon exhibited themselves, gave conclusive evidences of the formation of an abscess.

Although at this particular stage of this very critical disease, the work of the Surgeon is sometimes called into requisition, the united judgment of him and the physician is often insufficient to determine the proper course to be pursued. If evacuation of the pus externally is sought before adhesion to the walls of the abdomen has occurred, no good is effected, but on the contrary, the dissolution of the patient is hastened. On the other hand, if the puncturing is delayed too long, there is danger of the contents of the abscess making their way in a direction to favor the rupture of the peritoneum, and a discharge into the cavity.

Unfortunately, there is no certain symptom by which it can be known that perfect adhesion has taken place. It is only by the general appearance and prominence of the tumor that we form our conclusions.

In this case was verified a theory of Dysentery, which, though not universally applicable, is often correct in Sporadic cases of this painful affection of the bowels—we mean the theory of portal obstruction. A constant tendency to dysentery existed most of the time, and it is but natural to conclude that, in this case at least, it was the result of obstruction to the venous circulation through the liver.

The principal part of the treatment consisted in a succession of blisters over the affected part. This, together with a few opiates shortly after taken under treatment, relieved almost entirely the cough, which at the first was so distressing.

There being at no time the slightest evidence of attachment of the affected organ to the parietes of the abdomen, no hope could be indulged of benefit from the bistoury; therefore, no attempt was made to discharge the pus externally. And notwithstanding, in anticipation of the internal discharge of the pus, perfect quiet was enjoined; finally, when the walls of the abscess could hold out no longer, a rupture occurred, and the contents discharged into the cavity of the peritoneum. A general enlargement of the abdomen ensued, and the patient already prostrated by the protracted disease, sunk in a few hours under the violence of the shock.

*Pectoral Diseases.*—Some important Pneumonic affections were presented for consideration. Among them, a case of habitual *Asthma*, in a lad ten or twelve years old, was before the Class. The disease manifested itself in early infancy, and there being a continual want of expansion of the lungs, considerable contraction and deformity of the chest was the result. The father of the boy was desirous to have him relieved; but from the hopelessness of the case, not sufficient encouragement could be offered to warrant his continuance in the Clinic.

A case of *Tuberculosis*, affecting the larynx, lungs, &c., was before the Class, and afforded an opportunity to witness the various abnormal sounds produced by extensive disease of the breathing apparatus. This subject, a negro woman, aged about twenty-eight, had been under our occasional observation for a year previously, and generally exhibited an anæmic unhealthy appearance; an occasional, but not violent, cough; a lethargic and generally depraved condition of vital energies. Notwithstanding, ordinarily, she was able to perform the usual duties of house-servant. Suddenly the cough became aggravated; uneasiness and pain in the chest came on, with febrile symptoms, &c., &c.

The suppuration and ulceration consequent upon this inflammatory state, in six or eight weeks, destroyed one (the left) entire lung, as proved by post mortem examination.

Notwithstanding the strong doubts entertained by some authors of the similarity of tuberculous disease to Scrofula, the history of this case, connected with that of the disease of her children, seems in some degree to favor the doctrine of their identity, admitting the generally conceded opinion of hereditary predisposition. Two of her children, a boy seven, and a girl five years old, exhibit indubitable evidences of confirmed Scrofula.

Although instances of this kind may be multiplied, in which tuberculous parents have Scrofulous children, yet an almost insuperable difficulty to those who advocate their identity, presents itself in the fact that the local deposit and consequent irritation of Phthisis Pulmonalis, is rarely seen in the subject of Scrofulous ulceration of the neck. It is nevertheless true that in the two conditions denominated Scrofula and Tuberculosis, the most prominent constitutional disturbance in both, is in the function of nutrition.

From our own observations, however, and from the observations of those who have had more extended opportunities of investigating this subject, we are forced to the conclusion, that there is transmitted from parent to child, and even to the second and third generation, a depraved state of the nutritive energies which dispose to these diseases; and that it may overleap an entire generation, appearing in the grand-child, while the own child may have escaped the hereditary taint. We are, also, from the case under consideration, and from others in recollection, brought to the conclusion, that the subject of this transmission may have symptoms either of Phthisis or Scrofula from the same hereditary constitution.

A very important feature connected with the study of these diseases, is the tuberculous deposits in the air passages. This was prominently so in the case under consideration. Even the larynx was not exempt from them, in consequence of which the voice, toward the termination of the disease, was almost destroyed. This part of the respiratory tube being often seriously affected in tuberculous disease, error in diagnosis frequently occurs; and fallacious hopes, even by the attending physician, are indulged. With what confidence does he seize the cauterizing probang, thinking he has to contend with a simple chronic laryngitis, and that only a few touches will be sufficient to arrest its progress. Vain hope! No sooner has the more violent irritation subsided in the parts to which the application was made, than the same state is found to exist in locations beyond the reach of the probang. He, not till then, is apprised that the disease is not local, but constitutional in its character. Then it is he ceases to promise speedy recovery, or no recovery at all.

It is a fact also, which we have observed, that the irritation, excitement and ulceration, set up in the larynx and trachea, by the deposit of miliary tubercles, are sometimes manifested previous to any irritation in the bronchial, or parenchymatous structure of the lungs. This being the case, it is not surprising that the physician is often ignorant of the true character of the affection, and treats it as a local laryngeal inflammation. There is no doubt that the lungs, in many cases, perform their functions properly for months, and even years sometimes, after the excitement is set up in the throat, from the very same cause which has existed in the vesicular and bronchial structure of the lungs the whole time. The irritation may commence in all these parts simultaneously ; or it may be, that the bronchia exhibit signs of local disease first; but generally, when the air tubes, as well as the lungs themselves, are the seats of this deposit—the larynx, trachea, bronchia, and parenchyma, successively, in the order in which they are here named, are the subjects of derangement.

